# Antibacterial and antibiotic-modifying activities of fractions and compounds from *Albizia adianthifolia* against MDR Gram-negative enteric bacteria

**DOI:** 10.1186/s12906-019-2537-1

**Published:** 2019-06-06

**Authors:** Cedric F. Tchinda, Gaiëlle Sonfack, Ingrid K. Simo, İlhami Çelik, Igor K. Voukeng, Blaise K. Nganou, Gabin T. M. Bitchagno, Sultan Funda Ekti, Mathieu Tene, Pierre Tane, Veronique P. Beng, Victor Kuete

**Affiliations:** 1 Department of Biochemistry, Faculty of Science, University of Dschang, P.O. Box 67, Dschang, Cameroon; 2 Department of Biochemistry, Faculty of Science, University of Yaounde I, Yaounde, Cameroon; 3 Department of Chemistry, Faculty of Science, University of Dschang, Dschang, Cameroon; 4 Department of Chemistry, Faculty of Science, Eskişehir Technical University, 26470 Eskişehir, Turkey

**Keywords:** *Albizia adianthifolia*, Antibiotic modifying activity, Fabaceae, Multi-drug resistance, Phytochemicals

## Abstract

**Background:**

*Albizia adianthifolia* (Schum.) is medicinally used in Cameroon to manage bronchitis and skin diseases. Our previous study documented the antibacterial potential of its roots’ methanol extract. In this study, methanol roots extract was subjected to chromatography techniques and fractions (AARa and AARb), sub-fractions (AARa1–4, AARb1–2 and AARb11–14) together with isolated phytochemicals were assessed for their antimicrobial as well as their antibiotic-potentiating effects towards Gram-negative multidrug resistant (MDR) bacteria.

**Methods:**

The antibacterial activities of the samples (determination of Minimal Inhibitory « MIC » and Minimal Bactericidal Concentration « MBC ») were determined by the modified rapid p-iodonitrotetrazolium chloride (INT) colorimetric assay, as well as those of antibiotics in association with the compounds. Column chromatography was applied to isolate phytochemicals from roots extract and their chemical structures were determined using spectroscopic techniques.

**Results:**

The phytochemicals isolated were stearic acid (**1**), a mixture (1:1) of stigmasterol and *β*-sitosterol (**2 +  3**), *β*-sitosterol 3-*O*-*β*-_D_-glucopyranoside (**4**), palmatin (**5**), homomangiferin (**6**) and mangiferin (**7**). Fraction AARa exhibited selective inhibitory effects whilst all tested bacteria were inhibited by AARb in MIC ranges of 8 to 1024 μg/mL. Sub-fractions AARb1–2 had MIC values between 8 μg/mL and 1024 μg/mL on all tested bacteria. Phytochemicals **4, 2 +  3** and **7** inhibited the growth of 54.54% (6/11), 45.45% (5/11) and 27.27% (3/11) tested bacterial strains, respectively. When tested with an efflux pumps inhibitor (Phenylalanine-Arginine-*β*-Naphthylamide or PAβN), the inhibitory effects of compounds **2 + 3** and **4** increased towards all the tested bacteria. In association with erythromycin (ERY), streptomycin (STR) and tetracycline (TET), compounds **2 + 3** and **4** had the most significant synergistic activity on the seven selected bacteria.

**Conclusion:**

The present study provides information on the possible use of *Albizia adianthifolia* and its constituents in the control of Gram-negative infections including MDR phenotypes.

**Electronic supplementary material:**

The online version of this article (10.1186/s12906-019-2537-1) contains supplementary material, which is available to authorized users.

## Background

Bacteria infectious still constitute a serious health concern worldwide and is responsible for the high morbidity and mortality. In spite of the progress achieved by pharmaceutical industries in the synthesis of new antibacterial agents in recent years, the resistance to available drugs remains a major problem globally [1]. Besides, the continuous emergence of multi-resistant bacteria considerably reduces the efficiency of antibiotics, increases the frequency of therapeutic failures and incurs economic burden, all of this in association with undesired side effects of synthetic antibiotics makes the fight against bacterial infection complicated [2, 3]. The resistance of these bacteria to the antimicrobial agents can be associated to the presence of membrane transporting systems called efflux pumps that would be responsible for the over expression of the multi-resistance phenomenon [4]. It is worth noting that among Gram-negative bacteria, the effect of the combination of efflux pumps and the reduction of membrane permeability is responsible for the high resistance against antibiotics often associated to these groups of organisms [5]. Among the Gram-negative bacteria, those presenting multi-resistance phenotype belong mostly to the RND (Resistance Nodulation-Cell division) family which is a tripartite efflux pump. The increasing multi-drug resistance (MDR) and the lack of novel antibiotics propel the research of new antibacterial agents from medicinal plants. This is especially prominent as plants and their derived substances have long been used by humans for medicinal purposes [6]. Today, it is estimated that about 80% of the world’s population have integrated the use of medicinal plant as primary healthcare modality [7]. Recently, several bioactive compounds have been reported to fight against MDR bacteria [8]. Some examples include *Paullinia pinnata* [9, 10], *Combretum mole* [11] and *Harungana madagascariensis* [12]*.* In our continuous endeavors to identify antibacterial agents from plants traditionally used to fight microbial infection targeted *Albizia adianthifolia* (Schum.) (Fabaceae). The plant is used in traditional medecine to treat skin diseases, bronchitis, inflamed eyes, tapeworm, headaches and sinusitis [13, 14]. In earlier studies on this plant, adianthifoliosides A, B and D [15, 16], lupeol*,* aurantiamide acetate [17] and prosapogenins [18] were isolated. Previously, we demonstrated the antibacterial activity of the methanol extract from the roots (AAR) [19]. Herein, a bioassay guided fractionation was conducted for in-depth analysis of the antibacterial as well as antibiotic-modulating effect of the methanol extract from the roots of *Albizia adianthifolia*.

## Methods

### General procedure

The spectrometers were used to register the high resolution mass spectra (HRMS) (Shimadzu hybrid LC-MS-IT-TOF) and NMR Spectra (Agilent DD2 NMR (400 MHz) spectrometer). The silica gel Merck 60 F_254_ [(0.2–0.5 mm) and (0.2–0.063 mm)] 70,230 and 230–400 mesh (Darmstadt, Germany) was used in column chromatography (CC) while pre-coated silica gel 60 F_254_ was used to analyze on thin layer chromatography (TLC) plates (Merck, Germany). The TLC was revealed with 20% sulphuric acid (H_2_SO_4_), heated at 100 °C.

### Plant material and extraction

The roots of *Albizia adianthifolia* were harvested in Mont Kala, Center Region (Cameroon) on April 2015. The botanical identification was confirmed by Dr. Marie Florence Sandrine Ngo Ngwe at the National herbarium of Cameroon (Yaoundé) by comparison with the voucher specimen available under the reference number 24729/SRF/Cam (roots, leaves, bark). No permission was necessary for sample’s collection. The powdered roots of *A. adianthifolia* (3000 g) were soaked in methanol (MeOH; 8 L) for 48 h. After filtration and removal of the solvent using a rotary evaporator under reduced pressure, 124 g of crude extract (AAR) was obtained.

### Isolation and purification of bioactive compounds from the roots extract of *A. adianthifolia*

A portion of AAR (122.50 g) was dissolved in water (100%), followed by liquid-liquid exhaustion in ethyl acetate (AcOEt). Two new fractions named AARa (36.50 g, EtOAc) and a AARb (82.5 g; residual portion) were obtained. Fraction AARb fraction (82.5 g) was further dissolved in water (100%), followed by liquid-liquid exhaustion in n-butanol (n-BuOH) to afford two sub-fractions named AARb1 (49.3 g; n-BuOH) and AARb2 a residual fraction (28.5 g).

Part of the fraction AARa (33.50 g) was subjected to silica gel column chromatography (CC) eluting with gradient of Hexane-EtOAc then EtOAc-MeOH. Sixty-one fractions of 300 mL each were collected and combined on the basis of their thin layer chromatography (TLC) profiles into four main fractions (frs) coded AARa1–4 [AARa1 (1–12, 4.80 g), AARa2 (13–30, 4.60 g), AARa3 (31–39, 4 g) and AARa4 (40–61, 8 g)]. Fraction AARa1 was filtered and washed with EtOAc to yield compound **1** as white powder (20 mg). Fraction AARa2 was filtered and washed with EtOAc to yield a mixture of phytosterols **2** and **3** (50 mg) as white powder. Fraction AARa4 (8 g) was subjected to silica gel CC eluting with a gradient of EtOAc-MeOH (100:0, 97: 3, 94: 6, 91: 9, 85: 15, 0: 100) affording six new sub-fractions (sub-frs) (AARa41-AARa46). Sub-fraction AARa41 was filtered and washed with ethyl acetate to yield compound **4** (25 mg) as a white powder. Sub-fractions AARa43 was further subjected to Sephadex LH-20 eluted with MeOH to yield compound **5** as a yellow powder (30 mg).

Part of the sub-fraction AARb1 (47 g) was subjected to silica gel CC eluting with gradient of EtOAc-MeOH. Ninety-two fractions of 300 mL each were collected and combined based on their TLC profiles into four main fractions coded AARb1–4 [AARb1 (1–12; 5.40 g), AARb2 (13–34; 8.50 g), AARb3 (35–76; 14.50 g) and AARb4 (77–92; 12.70 g)]. Fraction AARb3 (13 g) was subjected to silica gel column chromatography eluting with a gradient of EtOAc-MeOH (100: 0, 95: 5, 90: 10, 85: 15, 80: 20, 70: 30, 0: 100) affording five sub-fractions AARb31- AARb35. Sub-fraction AARb32 was further subjected to Sephadex LH-20 eluted with MeOH to yield compound **6** (20 mg) and compound **7** (25 mg) as yellow powder each. This procedure of purification was bio-guided by antibacterial activity.

#### Antibacterial assays

### Chemicals for antibacterial assays

In this study, reference antibiotics used included: chloramphenicol (CHL), ciproflocaxin (CIP), erythromycin (ERY), gentamycin (GEN), kanamycin (KAN), norflocaxin (NOR), penicillin G (PEN), streptomycin (STR), and tetracycline (TET) obtained from Sigma-Aldrich (St Quentin Fallavier, France). Dimethyl sulfoxide (DMSO, Sigma-Aldrich) was used to dissolve the tesyed samples. The microbial growth indicator used was *p*-iodonitrotretrazolium chloride ≥97% (INT, Sigma-Aldrich) while the Efflux Pump Inhibitor (EPI) used was phenylalanine-arginine-*β*-naphthylamide (PAβN).

### Bacterial strains and culture media

A panel of 15 Gram-negative bacteria were investigated in this work. They included resistant strains of *Escherichia coli*, *Enterobacter aerogenes*, *Klebsiella pneumoniae*, *Providencia stuartii* and *Pseudomonas aeruginosa.* The bacteria strains used in this study were obtained both from the American Type Culture Collection (ATCC) or were clinical Laboratory isolates. Their bacterial characteristics were earlier given (Additional file 1; Table S1) [10]. Prior to the test, bacteria were cultured on Mueller Hinton Agar (MHA; Sigma) slant meanwhile Mueller Hinton Broth (MHB; Sigma) was used for antibacterial assay [20].

### Antibacterial testing

The minimum inhibitory concentration (MIC) of samples was evaluated following the broth microdilution using the well-known rapid INT method [21, 22]. Fractions, compounds and reference drug were dissolved in DMSO-MHB. The bacterial inoculum used was 1.5 × 10^6^ CFU/mL and the incubation conditions at 37 °C and 18 h. DMSO at less than 2.5% was used as solvent control while CHL was used as positive control.

Six isolated compounds were tested in the presence of an efflux inhibitor (EPI), PAβN (at 30 μg/mL) against ten bacteria including resistant strains in order to evaluate the role of efflux pumps in their resistance ability.

A preliminary assay was performed by assessing a combination of isolated phytochemical (**2 + 3**) at its various sub-inhibitory concentration and antibiotic on PA124 (see Additional file1; Table S3) which permitted us selecting appropriate sub-inhibitory concentration for further potentiating effect on other bacteria. Therefore, MIC/2 and MIC/4 were subsequently used for sample-antibiotics combination on more bacteria [6, 9, 23, 24].

Fractional inhibitory concentrations were calculated as the ratio of MIC of antibiotic in the combination, to that of the antibiotic alone (MIC_Antibiotic in combination_/MIC_Antibiotic alone_) and the interpretation done thus; Synergistic (≤ 0.5), Indifferent (1 to 4), or antagonistic (> 4) [25, 26].

## Results

### Phytochemicals

**Fig. 1:**
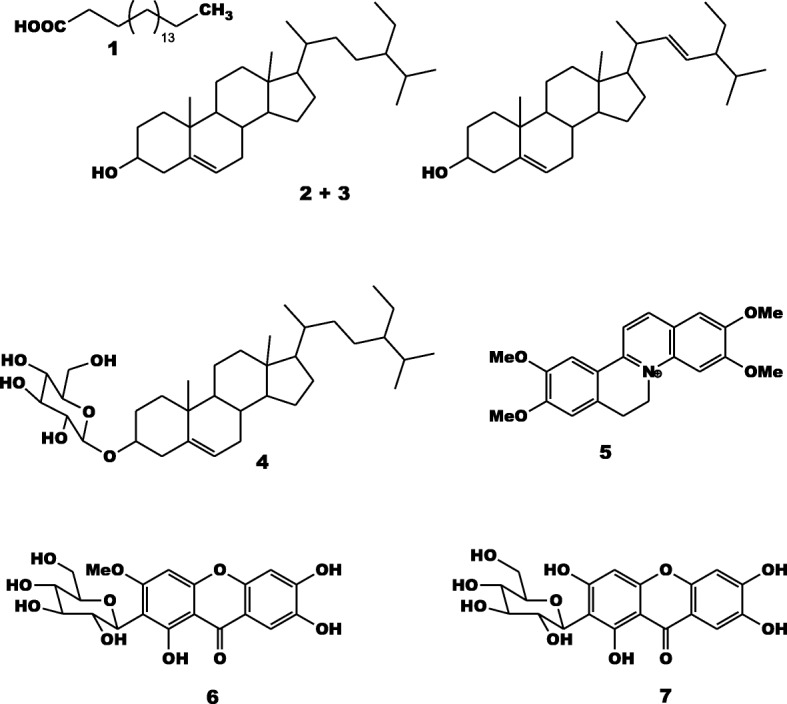
Chemical structures of compounds isolated from the roots of *Albizia adianthifolia*. Stearic acid (**1**), mixture of stigmasterol and *β*-sitosterol (**2 + 3**), *β*-sitosterol 3-*O-β*-_D_-glucopyranoside (**4**), palmatin (**5**), homomangiferin (**6**) and mangiferin (**7**)

The chemical structures of compounds (Fig. 1) namely stearic acid C_18_H_36_O_2_ (**1**, *m/z* 284, m.p.: 68–70 °C) [27], mixture (1:1) of stigmasterol and *β*-sistosterol (**2 + 3**) [28], *β*-sitosterol 3-*O*-*β*-_D_-glucopyranoside C_35_H_60_O_6_ (**4**, *m/z* 576) [29], palmatin C_21_H_22_NO_4_^+^ (**5**, *m/z* 352, m.p.: 204–206 °C) [30], homomangiferin C_20_H_20_O_11_ (**6**, *m/z* 436, m.p.: 249–251 °C) [31] and mangiferin C_19_H_18_O_11_ (7, *m/z* 422, m.p.: 259–260 °C) [32], from *A. adianthifolia* roots extract, were determined using physical and NMR (^1^H, ^13^C and 2D) data, in comparison with those of related compounds in the literature (Additional file 1).

### Antibacterial activity

The inhibitory potential towards 15 Gram-negative bacteria of fractions (AARa-b), sub-fractions fractions (AARa1–4, AARb1–2 and AARb11–14) as well as phytochemicals from the roots of *A. adianthifolia*, and CHL is given in Tables 1 and 2. It appears from data in Table 1 that the tested botanicals (crude extract, fractions and sub-frs) and phytochemicals were selectively active. The recorded MIC values were in the range of 8 to 1024 μg/mL. However, fraction AARb was active on 15 of the 15 (100%) bacteria tested, while AARa was active on 73.33% (11/15) of them. MICs ≤256 μg/mL were obtained with CHL on 100% (15/15) of the bacteria tested. MBC ≤ 1024 μg/mL were noted with AARa-b on some of the studied bacteria. Table 1 shows the MICs and MBCs of AARa sub-frs (AARa1–4) on the panel of 15 bacteria. As a result, the AARa2 and AARa3 sub-frs had MICs ranged from 16 and 1024 μg/mL on all tested pathogens contrary to other sub-frs showed selective activities. These inhibitory activities were observed on 68.66% (13/15), 80% (12/15), 40% (6/15) and 33.33% (5/15) bacteria tested with the sub-fractions AARa2, AARa3, AARa4 and AARa1 respectively. MICs and MBCs as seen in Table 1 for AARb sub-fractions (AARb1–2) on the panel of 15 bacteria indicated that AARb1–2 had MICs ranged from 8 to 1024 μg/mL on all the tested bacteria. They were active on 93.33% (14/15) of the tested bacteria. The investigation of sub-fractions of AARb11-AARb14 is summarized in Table 1 as well. MICs varying from 8 to 1024 μg/mL were obtained and the recorded inhibitory effects were noted on 100% (15/15), 93.33% (14/15), 80% (12/15) and 60% (9/15) of the bacteria tested with AARb13, AARb14, AARb11 and AARb12 respectively. In general, the MBCs were above 1024 μg/mL.

**Table 1:** MIC and MBC (in μg/mL) of fractions, sub-fractions of *A. adianthifolia* roots and chloramphenicol againts the panel of 15 Gram-negative bacteria

Bacterial strains		Tested samples, MIC and MBC in parenthesis (in μg/mL)												
		Fractions		Fractions and sub-fractions										CHL
		AARa	AARb	AARa1	AARa2	AARa3	AARa4	AARb1	AARb2	AARb11	AARb12	AARb13	AAR14	
*E. coli*	AG100Atet	1024(−)	64(512)	1024(−)	512(−)	512(−)	1024(−)	64(512)	64(−)	512(−)	1024(−)	64(−)	256(−)	32(256)
	AG102	-(−)	256(1024)	-(−)	512(−)	512(−)	1024(−)	128(−)	512(−)	1024(−)	-(−)	128(1024)	256(−)	32(256)
	ATCC8739	512(−)	256(−)	-(−)	512(−)	512(1024)	1024(−)	16(256)	64(512)	64(1024)	128(−)	16(1024)	16(1024)	2(64)
	ATCC 10536	1024(−)	128(1024)	-(−)	512(1024)	512(1024)	1024(−)	32(1024)	64(1024)	128(−)	256(−)	32(−)	64(−)	2(32)
*E. aerogenes*	ATCC13048	512(1024)	128(1024)	-(−)	256(1024)	256(−)	–	16(512)	32(1024)	128(−)	512(1024)	64(256)	128(512)	16(128)
	CM64	1024(−)	256(1024)	-(−)	256(512)	512(1024)	–	32(−)	64(−)	256(1024)	512(−)	32(128)	256(1024)	256(−)
	EA27	512(−)	8(128)	1024(−)	32(512)	16(512)	128(512)	32(−)	64(−)	128(−)	-(−)	32(1024)	128(−)	32(256)
	EA289	-(−)	256(1024)	-(−)	-(−)	-(−)	-(−)	32(−)	64(−)	512(−)	-(−)	64(−)	128(−)	32(256)
*K. pneumoniae*	ATCC11296	1024(−)	256(−)	-(−)	1024(−)	-(−)	-(−)	16(−)	64(−)	128(−)	512(−)	32(512)	32(256)	32(256)
	KP55	256(−)	128(−)	-(−)	256(−)	256(−)	-(−)	16(1024)	32(−)	128(−)	256(−)	16(512)	32(1024)	64(256)
	KP63	256(−)	128(−)	512(1024)	256(−)	512(−)	1024(−)	8(512)	16(−)	128(−)	128(1024)	8(128)	128(−)	32(256)
*P.* *stuartii*	ATCC29916	-(−)	128(1024)	1024(−)	512(−)	512(1024)	-(−)	128(−)	256(−)	-(−)	-(−)	256(−)	512(−)	64(256)
	NEA 16	1024(−)	256(1024)	1024(−)	-(−)	-(−)	-(−)	128(−)	256(−)	-(−)	-(−)	128(1024)	256(−)	64(256)
*P. aeruginosa*	PA01	1024(−)	256(−)	-(−)	512(−)	512(−)	-(−)	64(−)	64(−)	256(−)	512(−)	32(−)	64(−)	64(−)
	PA124	-(−)	256(−)	-(−)	512(−)	512(−)	-(−)	-(−)	-(−)	-(−)	-(−)	1024(−)	-(−)	256(−)

Tested samples were ethyl acetate fraction (AARa), residual ethyl acetate fraction (AARb), sub-fractions of ethyl acetate fraction (AARa1–4), sub-fractions of residual ethyl acetate fraction (n-butanol fraction « AARb1 » and residual n-butanol fraction «AARb2»), sub-fractions of n-butanol fraction (AARb11–14) and chloramphénicol (CHL); −: MIC or MBC values above 1024 μg/mL

**Table 2:** MIC and MBC (in μg/mL) of compounds isolated from *A. adianthifolia* roots againts the panel of 11 Gram-negative bacteria

Bacterial strains	Compounds, MIC and MBC in parenthesis (in μg/mL)					
	2+ 3	4	5	6	7	CHL
*E. coli*						
AG102	–	128(−)	–	–	–	32(−)
ATCC8739	–	16(32)	–	–	–	2(64)
ATCC 10536	16(32)	16(32)	–	32(−)	64(−)	2(32)
*E. aerogenes*						
ATCC13048	128 (−)	128(−)	128(−)	128(−)	128(−)	16(−)
EA27	–	–	–	–	–	32(−)
*K. pneumoniae*						
ATCC11296	–	–	128(−)	–	–	32(−)
KP55	32(64)	–	128(−)	128 (−)	128(−)	64(−)
*P. stuartii*					**–**	
ATCC29916	64(128)	–	64(128)	128 (−)	–	64(−)
NEA 16	–	–	–	–	–	64(−)
*P. aeruginosa*						
PA01	16(64)	2(64)	–	–	–	64(−)
PA124	–	128(−)	–	–	–	–

**-:** MIC or MBC values above 128 μg/mL; compound **1** was not active at up to 128 μg/mL

The antibacterial activity of compounds isolated from the roots of *A. adianthifolia* is compiled in Table 2. Compounds **4, 2 + 3** and **7** respectively inhibited the growth of 54.5% (6/11), 45.4% (5/11) and 27.3% (3/11) of tested bacteria, whereas compounds **5** and **6** exhibited similar activities by inhibiting each 36.7% (4/11) bacteria tested. The activity of the compound **(2 + 3)** vis-à-vis *K. pneumoniae* KP55 (MIC of 32 μg/mL); compounds **2 + 3** and **4** vis-a-vis *P. aeruginosa* PA01 (MIC of 16 μg /mL and MIC of 2 μg/mL respectively) and compound **4** vis-à-vis *P. aeruginosa* PA124 (MIC of 128 μg/mL) were greater compared to that of CHL. At a concentration as high as 128 μg/mL, compound **1** had no antibacterial activity. The bactericidal effect of **2 + 3, 4** and **5** were noted vis-à-vis 3/11, 2/11 and 1/11 pathogens tested respectively.

### Influence of the bacterial efflux pumps on the activity of the tested phytochemicals

Ten selected MDR bacteria were tested in the presence of EPI (PAβN). It appears that in the combination with PAβN, the activities of compounds **2 + 3** and **4** were ameliorated against 100% (10/10) of tested MDR strains (Table 3) while the other compounds (**5, 6** and **7**) displayed moderate activity in the presence of EPI.

**Table 3:** MIC in μg/mL of compounds and chloramphenicol in the presence of PAβN

Bacterial strains		Tested samples, MIC alone, MIC in the present of PAβN (μg/mL), and ameliorating factor (FA)																	
		2 + 3			4			5			6			7			CHL		
		MIC	+PAβN	FA	MIC	+PAβN	FA	MIC	+PAβN	FA	MIC	+PAβN	FA	MIC	+PAβN	FA	MIC	+PAβN	FA
*E. coli*	AG102	–	128	> 1	128	64	2	–	–	–	–	–	–	–	–	–	32	4	8
	ATCC10536	16	4	4	16	8	2	–	128	> 1	32	8	4	64	8	8	2	< 1	< 2
*E. aerogenes*	ATCC13048	128	64	2	128	32	4	128	64	2	128	64	2	128	32	4	16	8	2
	EA27	–	128	> 1	–	128	> 1	–	–	–	–	–	–	–	–	–	32	16	2
*K. pneumoniae*	ATCC11296	–	128	> 1	–	128	> 1	128	32	4	–	–	–	–	–	–	32	8	4
	KP55	32	8	4	–	128	> 1	128	64	2	128	128	1	128	16	8	64	32	2
*P. stuartii*	ATCC29916	64	2	32	–	16	> 8	64	16	4	128	32	4	–	–	–	64	8	8
	NEA16	–	8	> 16	–	16	> 8	–	128	> 1	–	128	> 1	–	128	> 1	64	16	4
*P. aeruginosa*	PA01	16	8	2	2	< 1	> 2	–	–	–	–	–	–	–	–	–	64	8	8
	PA124	–	2	> 64	128	64	2	–	128	> 1	–	–	–	–	–	–	256	16	16

*CHL* chloramphenicol, *PAßN* Phenylalanine arginyl ß-Naphtylamide. Ameliorating factor: correspond to the ratio MIC of sample tested alone/ MIC of sample in presence of PAßN, −**:** > 1024 μg/mL (case of crude extract), −**:** > 128 μg/mL (case of compounds). PAßN was tested at 30 μg/mL

### Potentiating effect of phytochemicals

Based on results obtained from a preliminary study carried out on *Pseudomonas aeruginosa* PA124, three isolated molecules were associated with seven antibiotics (CIP, ERY, GEN, KAN, NOR, STR, and TET) to ascertain the ability to potentiate their activities. Tables 4 and 5 show synergies between phytochemicals and the majority of antibiotics. These synergistic effects varied from 28.57 to 100% on the various microorganisms with all the compounds. In combination with ERY and STR antibiotics, all compounds **2 + 3** and **4** showed the most significant synergistic effects (100%) at their different sub-inhibitory concentrations (MIC/2 and MIC/4). Besides, these samples, namely compounds **2 + 3** and **4** in association with KAN, presented the weakest synergistic effects, ranging from 28.57 to 71.42% compared to the other antibiotics of the panel used. The synergistic effect was also noted (100%) with compounds **2 + 3** and **4** in combination with TET against the tested bacteria (Table 4); this was also the case when compound **4** (at MIC/2) was combined with GEN (Table 5). No antagonistic effect was noted when compounds were combined with antibiotics. However, indifference effects were observed in some cases.

**Table 4:** MIC of antibiotics after the association with compound 2 + 3 at MIC/2 and MIC/4 against seven MDR bacteria strains

Antibiotics^a^	Bacterial strains^b^, MIC (μg/mL) of antibiotics in the absence and presence of compound 2 + 3								
	Compounds concentration	PA124	KP55	ATCC11296	EA27	ATCC13048	AG102	ATCC10536	PBSS (%)
CIP	0	2	0.5	0.5	0.5	4	2	0.125	
	CMI/2	0.5(0.25)S	0.5(1)I	0.25 (0.5)S	0.25 (0.5)S	0.125(0.031)S	0.5(0.25)S	0.125 (1)I	(5/7) 71.42%
	CMI/4	0.5(0.25)S	0.5 (1)I	0.25 (0.5)S	0.5 (1)I	0.5 (0.125)S	1 (0.5)S	0.125 (1)I	(4/7) 57.14%
ERY	0	> 32	4	> 32	> 32	> 32	16	16	
	CMI/2	32(0.5)S	2(0.5)S	4(< 0.125)S	32 (0.5)S	32 (0.5)S	8 (0.5)S	8 (0.5)S	(7/7) 100%
	CMI/4	32(0.5)S	2(0.5)S	4(< 0.125)S	32 (0.5)S	32 (0.5)S	8 (0.5)S	8 (0.5)S	(7/7) 100%
GEN	0	> 4	2	> 4	4	4	> 4	4	
	CMI/2	4(0.5)S	1(0.5)S	0.125(< 0.031)S	4(1)I	2(0.5)S	4 (0.5)S	2(0.5)S	(6/7) 85.71%
	CMI/4	4(0.5)S	1(0.5)S	0.125(< 0.031)S	4(1)I	2(0.5)S	4 (0.5)S	2(0.5)S	(6/7) 85.71%
KAN	0	0.5	2	4	4	16	16	4	
	CMI/2	< 0.125(0.25)S	2(1)I	2(0.5)S	4(1)I	16(1)I	8 (0.5)S	4(1)I	(3/7) 42.85%
	CMI/4	< 0.125(0.25)S	2(1)I	2(0.5)S	4(1)I	16(1)I	8 (0.5)S	4(1)I	(3/7) 42.85%
NOR	0	> 16	16	1	16	16	2	1	
	CMI/2	< 0.125(0.007)S	8(0.5)S	1(1)I	4(0.25)S	2(0.125)S	2 (1)I	1 (1)I	(4/7) 57.14%
	CMI/4	< 0.125(0.007)S	8(0.5)S	1(1)I	4(0.25)S	4(0.25)S	2 (1)I	1 (1)I	(4/7) 57.14%
STR	0	> 32	> 32	> 32	> 32	> 32	> 32	> 32	
	CMI/2	32(0.5)S	16(< 0.5)S	32(0.5)S	16(< 0.5)S	32(0.5)S	16(< 0.5)S	2(< 0.062)S	(7/7) 100%
	CMI/4	32(0.5)S	32(0.5)S	32(0.5)S	16(< 0.5)S	32(0.5)S	16(< 0.5)S	4(< 0.125)S	(7/7) 100%
TET	0	8	0.125	> 16	> 16	> 16	> 16	16	
	CMI/2	4(0.5)S	0.0625(0.5)S	8(< 0.5)S	16(0.5)S	4(< 0.25)S	1(< 0.062)S	0.125(0.007)S	(7/7) 100%
	CMI/4	4(0.5)S	0.0625(0.5)S	8(< 0.5)S	16(0.5)S	4(< 0.25)S	1(< 0.062)S	0.125(0.007)S	(7/7) 100%

^a^Antibiotics [CIP: Ciprofloxacin, ERY: Erythromycin, GEN: Gentamycin, KAN: Kanamycin, NOR: Norfloxacin, STR: Streptomycin, TET: Tetracycline]. ^b^Bacteria: *Escherichia coli* [ATCC10536, AG102], *Pseudomonas aeruginosa* [PA124], *Enterobacter aerogenes* [ATCC13048, EA27], *Klebsiella pneumoniae* [ATCC11296, KP55]. PBSS: Percentage of bacteria strain on which synergism has been observed; S: Synergy; I: Indifference; (): FIC (Fractional Inhibitory Concentration) of the antibiotics after association with compounds, 0: MIC of the antibiotic alone

**Table 5:** MIC of different antibiotics after the association with compound **4** at MIC/2, MIC/4 against seven MDR bacteria strains

Antibiotics^a^	Bacterial strains^b^, MIC (μg/mL) of antibiotics in the absence and presence of compound 4								
	Compounds concentration	PA124	KP55	ATCC11296	EA27	ATCC13048	AG102	ATCC10536	PBSS (%)
CIP	0	2	0.5	0.5	0.5	4	2	0.125	
	CMI/2	2 (1)I	0.5(1)I	0.25(0.5)S	0.25(0.5)S	0.5(0.125)S	0.5(0.25)S	0.125(1)I	(4/7) 57.14%
	CMI/4	2 (1)I	0.5(1)I	0.25(0.5)S	0.25(0.5)S	0.5(0.125)S	0.5(0.25)S	0.125(1)I	(4/7) 57.14%
ERY	0	> 32	4	> 32	> 32	> 32	16	16	
	CMI/2	32 (0.5)S	2(0.5)S	4(< 0.125)S	32 (0.5)S	4(< 0.125)S	4 (0.25)S	8 (0.5)S	(7/7) 100%
	CMI/4	32 (0.5)S	2(0.5)S	4(< 0.125)S	32 (0.5)S	8(< 0.25)S	4 (0.25)S	8 (0.5)S	(7/7) 100%
GEN	0	> 4	2	> 4	4	4	> 4	4	
	CMI/2	4(0.5)S	1(0.5)S	0.0625(< 0.015)S	1 (0.25)S	2(0.5)S	4(0.5)S	0.5(0.125)S	(7/7) 100%
	CMI/4	4(0.5)S	2(1)I	0.0625(< 0.015)S	1 (0.25)S	(0.5)S	4(0.5)S	2(0.5)S	(6/7) 85.71%
KAN	0	0.5	2	4	4	16	16	4	
	CMI/2	< 0.125(0.25)S	1(0.5)S	2(0.5)S	4(1)I	16(1)I	16(1)I	4(1)I	(3/7) 42.85%
	CMI/4	< 0.125(0.25)S	2(1)I	2(0.5)S	4(1)I	16(1)I	16(1)I	4(1)I	(2/7) 28.57%
NOR	0	> 16	16	1	16	16	2	1	
	CMI/2	8(< 0.5)S	2(0.125)S	0.5(0.5)S	4(0.25)S	8(0.5)S	2(1)I	0.5(0.5)S	(6/7) 85.71%
	CMI/4	8(< 0.5)S	2(0.125)S	0.5(0.5)S	8(0.5)S	8(0.5)S	2(1)I	0.5(0.5)S	(6/7) 85.71%
STR	0	> 32	> 32	> 32	> 32	> 32	> 32	> 32	
	CMI/2	32(0.5)S	32(0.5)S	32(0.5)S	16(< 0.5)S	32(0.5)S	16(< 0.5)S	2(< 0.062)S	(7/7) 100%
	CMI/4	32(0.5)S	32(0.5)S	32(0.5)S	16(< 0.5)S	32(0.5)S	16(< 0.5)S	2(< 0.062)S	(7/7) 100%
TET	0	8	0.125	> 16	> 16	> 16	> 16	16	
	CMI/2	4(0.5)S	0.0625(0.5)S	0.5(< 0.031)S	4(< 0.25)S	0.5(< 0.031)S	2(< 0.125)S	0.125(0.007)S	(7/7) 100%
	CMI/4	4(0.5)S	0.0625(0.5)S	0.5(< 0.031)S	8(< 0.5)S	0.5(< 0.031)S	2(< 0.125)S	0.125(0.007)S	(7/7) 100%

^a^Antibiotics [*CIP* Ciprofloxacin, *ERY* Erythromycin, *GEN* Gentamycin, *KAN* Kanamycin, *NOR* Norfloxacin, *STR* Streptomycin, *TET* Tetracyclin]. ^b^Bacteria: *Escherichia coli* [ATCC10536, AG102], *Pseudomonas aeruginosa* [PA124], *Enterobacter aerogenes* [ATCC13048, EA27], *Klebsiella pneumoniae* [ATCC11296, KP55]. PBSS: Percentage of bacteria strain on which synergism has been observed; S: Synergy; I: Indifference; (): FIC (Fractional Inhibitory Concentration) of the antibiotics after association with compounds, 0: MIC of the antibiotic alone

## Discussion

### Phytochemicals

Several compounds (seven compounds) were identified in the present work, this include; fatty acid (1), mixture of steroids (2 + 3), one steroid glycoside (4), one alkaloid (5), and two xanthones (6, 7). The isolation of compounds such as adianthifoliosides (A, B and D), lupeol*,* aurantiamide acetate, prosapogenins from *Albizia adianthifolia* was published earlier [15–18]. Nonetheless, few phytochemicals were isolated herein. This could likely be because all fractions were not explored as the isolation procedure was biologically guided.

### Antibacterial effects

The need to search for new effective phytochemicals to combat MDR bacteria is timely. Thus, the activities of plant samples could be attributable to the presence of their phytochemical constituents [33, 34]. Previously we documented the antibacterial effects of crude extracts of *Albizia adianthifolia* leaves, bark and roots extracts [19]. This was the rationale for performing, in the present work, the bioguided purification of the roots extract. The inhibitory effect of the root extract of *Albizia adianthifolia* (AAR) was moderate [35], with MICs ≤625 μg/mL against various Gram-negative bacteria [19]. In the present study, fractionation of AAR afforded more effective fractions and sub-frs (Table 1). The recorded MIC values highlight the good activities of AARb vis-à-vis *E. coli* AG100Atet (64 μg/mL) and *E. aerogenes* EA27 (8 μg/mL), AARa2 and AARa3 against *E. aerogenes* EA27 (32 μg/mL and 16 μg/mL respectively), AARb1 and AARb2 against *K. pneumoniae* KP63 (8 μg/mL and 16 μg/mL respectively), AARb14 and AARb11 against *E. coli* ATCC8739 (16 μg/mL and 64 μg/mL respectively) and AARb13 against *K. pneumoniae* KP63 (8 μg/mL). This clearly demonstrates the increase in the activity related to the subsequent fractionation of the plant extract, thus reflecting the good antibacterial potential of *Albizia adanthifolia*. It should also be noted that AARb1 and AARb2 showed MICs < 100 μg/mL on the majority of bacteria (11/15) (Table 1). The MBC/MIC ratios obtained were generally greater than 4, highlighting the bacteriostatic effects of extracts studied as well as the active fractions [36, 37]. According to established cutoff points [38], the antibacterial activity of phytochemicals are significant when MICs < 10 μg/mL, moderate when MICs are between 10 and 100 μg/mL, and low if the MICs > 100 μg/mL. On the basis of this scale, compound **4** had significant antibacterial effects against *P. aeruginosa* PA01 (MIC of 2 μg/mL) strain. Overall MIC data obtained with the compounds were much higher than those of the most active sub-fractions from where they were isolated (AARb1–2 and AARb13). This suggests that the antibacterial activity of its sub-fractions could be due to the synergistic effects of its different constituents. This is also an indication that the fight against the pathogens tested with fractions, sub-fractions and mainly AARb13 (sub-fraction) could be more effective than with isolated compounds.

### Role of efflux pumps in the susceptibility of gram-negative bacteria to the tested samples

The efflux systems involved in this mechanism are tripartite complexes, including the AcrAB-TolC and MexAB-oprM pumps of Enterobacteriaceae and *P. aeruginosa* respectively [39, 40], which play a central role in the multidrug resistance of Gram-negative bacteria. The restoration of the sensitivity of bacteria by the use of efflux pumps inhibitors (IPE) to allow an achievement of the antibacterial threshold concentration, capable of inducing the death of the bacterial cell is the best-known way to combat this type of resistance. PAßN is a potent inhibitor of the aforementioned pumps [41]. In this study, it was found that compounds **2 + 3** and **4** showed an improvement in their activity in the presence of EPI on 100% of the pathogens used. These phytochemicals in combination with EPI could be used in the fight against bacterial infections due to multidrug-resistant phenotypes over-expressing active efflux pumps. The other compounds, namely, **5, 6** and **7**, which had a rather moderate improvement both in intensity and frequency, would be least affected by the effect of efflux pumps. The improvement of the activity of these substances (compounds) in the presence of PAβN could also be explained by a facilitation of the penetration of the active principles into the bacterial cells as previously demonstrated by Lamers et al. [42] with *P. aeruginosa.*

### Effects of association of compounds with antibiotics

Synergistic effects following the combination of the compounds (**2 + 3** and **4**) with ERY, STR, as well as with GEN and compounds **2 + 3** and **4** with TET with respect to all the bacteria tested were noted. Synergistic or modulatory effects of the compounds (**2 + 3** and **4**) with other antibiotics were found on more than 70% of bacteria tested in several cases, with FIC values, ranging mostly from 0.5 to 0.007. These results suggest that those compounds could be considered as a potential efflux inhibitor [23]. The antibacterial potential of compounds (**2 + 3** and **4**) in the inhibition of resistant Gram-negative bacteria is reported here for the first time, as well as their antibiotic-modulatory effects. This study also provides more information on the antibacterial activity of compounds (**2 + 3** and **4**) against MDR bacteria.

## Conclusion

Data reported in the present investigation suggest that bioactives from root of *Albizia adianthifolia* are potential sources of antibacterials to tackle resistant phenotypes. To overcome bacterial resistance, compounds **2 + 3** and **4** could also possibly be used in association with antibiotics.

## Additional file

Additional file 1: **Table S1.** Bacterial strains used and their features. **S2** ^**1**^H, 13C RMN and major chemical shifts of studied compounds. **Table S3** Preliminary evaluation of antibiotic-resistance modulatory activity of selected samples at sub-inhibitory concentrations against *Pseudomonnas aeruginosa PA124. (DOC 848 kb)*

## Data Availability

All data generated or analysed during this study are included in this published article and its Additional files.

## Abbreviations

AAR: Methanol extract from the roots of *Albizia adianthifolia*
AARa-b: fractions from AAR
ATB: Antibiotic
ATCC: American Type Culture Collection
CC: Column chromatography
CFU: Colony Forming Unit
CHL: Chloramphenicol
CIP: Ciprofloxacin
DMSO: Dimethyl sulfoxide
*E. aerogenes*: *Enterobacter aerogenes*
*E. coli*: *Escherichia coli*
ERY: Erythromycin
EtOAc: Ethyl acetate
GEN: Gentamycin
INT: p-iodonitrotetrazolium chloride ≥97% (INT, Sigma-Aldrich)
*K. pneumoniae*: *Klebsiella pneumoniae*
KAN: Kanamycin
MBC: Minimal Bactericidal Concentration
MDR: Multidrug resistant
MeOH: Methanol
MHB: Mueller Hinton Broth
MIC: Minimal Inhibitory Concentration
NOR: Norflocaxin
*P. aeruginosa*: *Pseudomonas aeruginosa*
*P. stuartii*: *Providencia stuartii*
PEN: Penicillin
STR: Streptomycin
TET: Tetracyclin
TLC: Thin Layer Chromatography
AARb1–2: Sub-fractions from AARb
AARa1–4: Sub-fractions from AARa
