# Supplementary material for: Antibacterial and antibiotic-modifying activities of fractions and compounds from Albizia adianthifolia against MDR Gram-negative enteric bacteria
Source: BMC Complement Altern Med. 2019 Jun 6;19:120. doi: 10.1186/s12906-019-2537-1 (PMC6554897; doi:10.1186/s12906-019-2537-1)
Supplement: Supplementary file 1 — Table S1. Bacterial strains used and their features. S2 1H, 13C RMN and major chemical shifts of studied compounds. Table S3 Preliminary evaluation of antibiotic-resistance modulatory activity of selected samples at sub-inhibitory concentrations against Pseudomonnas aeruginosa PA124. (DOC 848 kb) [file 12906_2019_2537_MOESM1_ESM.doc]

**Antibacterial and antibiotic-** **modifying activities of fractions and compounds from *Albizia adianthifolia* against MDR Gram-negative enteric bacteria**

Cedric F. Tchinda^1,2^, Gaiëlle Sonfack^3^, Ingrid K. Simo^3^, İlhami Çelik^4^, Igor K. Voukeng^1^, Blaise K. Nganou^3^, Gabin T.M. Bitchagno^3^, Sultan Funda Ekti^4^, Mathieu Tene^3^, Pierre Tane^3^, Veronique P. Beng^2^, and Victor Kuete^1*^

*^1^Department of Biochemistry, Faculty of Science, University of Dschang, Dschang, Cameroon;*

*^2^Department of Biochemistry, Faculty of Science, University of Yaounde I, Yaounde, Cameroon;*

*^3^Department of Chemistry, Faculty of Science, University of Dschang, Dschang, Cameroon;*

*^4^Department of Chemistry, Faculty of Science, Eskişehir Technical University, 26470 Eskişehir, Turkey*

***Corresponding author:

*Tel : (237) 77 35 59 27; P.O. Box 67 Dschang, Cameroon; E-mail:* [*kuetevictor@yahoo.fr*](mailto:kuetevictor@yahoo.fr) *(Prof. Victor Kuete)*

**S1.** Bacterial strains and features

| **Espèces** | **Souches** | **Caractéristiques** | | **Références** |
| --- | --- | --- | --- | --- |
| ***Escherichia***  ***Coli*** | ATCC8739 | Reference strain | |  |
|  | ATCC10536 | Reference strain | |  |
|  | AG 100ATet | ΔacrAB mutant AG 100A Tet^r^ | | [1] |
|  | AG 102 | AG 100 expressing *Acr AB* pumps | | [2] |
|  |  |  |  |  |
|  |  |  |  |  |
| ***Enterobacter aerogenes*** | ATCC13048 | Reference strain | |  |
|  |  |  |  |  |
|  | CM64 | CHL^R^ resistant strain ATCC13048 over expressing AcrAB pumps | | [3, 4] |
|  | EA27 | Clinical MDR isolate exhibiting  energy-dependent norfloxacin and  chloramphenicol efflux with KAN^R^ and  AMP^R^ and NAL^R^ and STR^R^ and TET^R^ | | [3, 4] |
|  | EA 289 | KAN sensitive derivative of EA27 | | [5] |
|  |  |  |  |  |
|  |  |  |  |  |
| ***Klebsiella pneumoniae*** | ATCC11296 | Reference strain | |  |
|  |  |  |  |  |
|  | KP55 | Clinical MDR isolate, TET^R^, AMP^R^, ATM^R^, and CEF^R^ | | [6] |
|  | KP63 | Clinical MDR isolate, TET^R^, CHL^R^, AMP^R^, and ATM^R^ | | [6] |
| ***Providencia stuartii*** | ATCC 29916 | Reference strain | |  |
|  | NEA16 | Clinical MDR isolate of *Providencia stuartii* expressing *AcrAB-TolC* | | [7] |
|  |  |  |  |  |
| ***Pseudomonas aeruginosa*** | PA01 | Reference strain | |  |
|  | PA124 | MDR clinical isolate *expressing MexAB-OprMpump* | | [8] |

AMP^r^, ATM^r^, CEF^r^, CHL^r^, KAN^r^, NAL^r^, NOR^r^, STR^r^ et TET^r^, resistance to:ampicilline, aztreonam, céfépime, chloramphenicol, kanamycine, acidenalidixique, norfloxine, streptomycine and tétracycline respectively ;*AcrAB-TolC, MexAB-OprM :* pumps efflux.

**S2. RMN ^1^H, 13C and major chemical shifts of studied compounds**

- **Compound 1 (Stearic acid)**

**Stearic acid,** C_18_H_36_O_2_; (m/z 284). White powder. ^13^C NMR (CDCl_3_, 100 MHz) *δ* (ppm): 179.3 (C-1), 33.9 (C-2), 31.9-24.7 (Aliphatic methylene), 24.7 (C-3), 14.3 (C-18). ^1^H NMR (CDCl_3_, 400 MHz) *δ* (ppm) : 2.34 (2H, t, H-2, *J* =7.5), 1.61 (1H, m, H-3), 1.50-1.10 (Aliphatic methylene), 0.86 (3H, t, H-18).


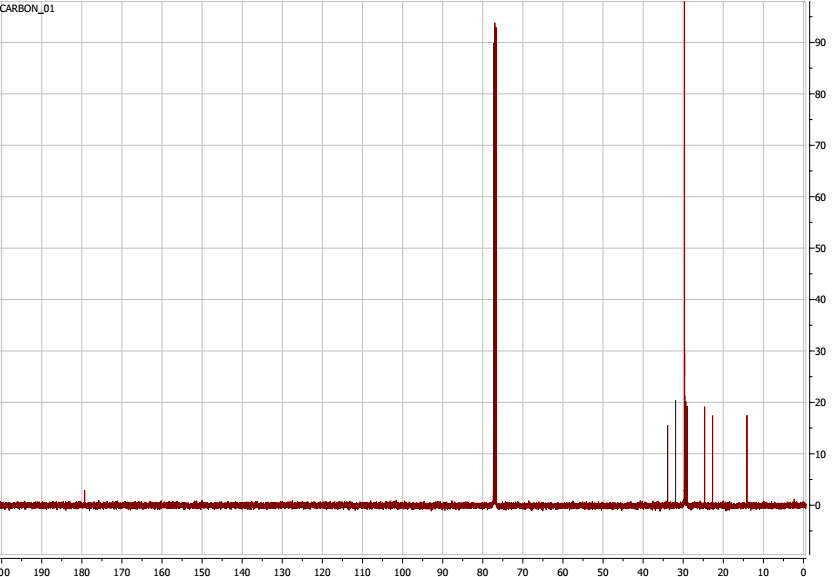


^13^C RMN (CDCl_3_, 100 MHz) of compound **1**


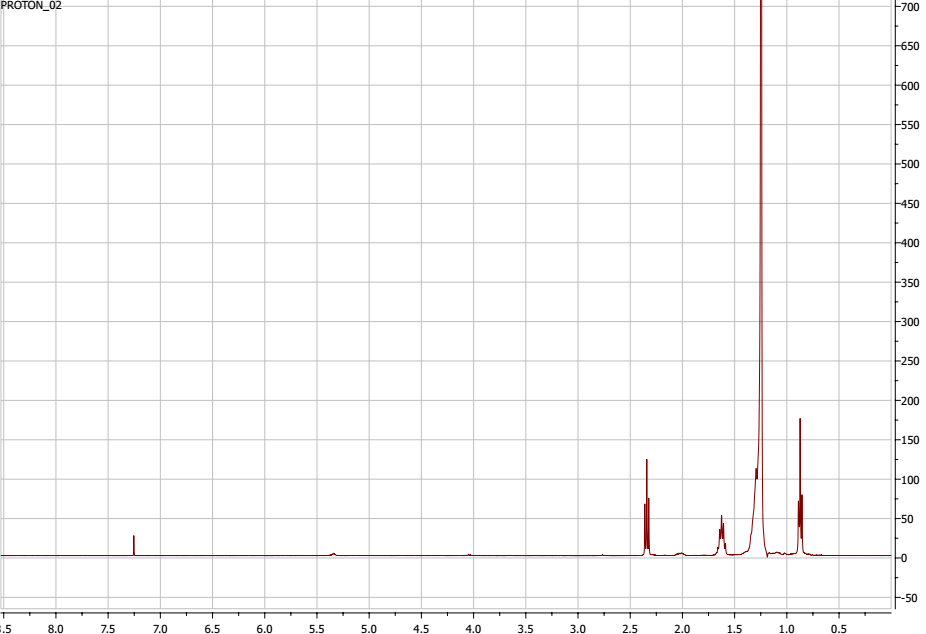


^1^H RMN (CDCl_3_, 400 MHz) of compound **1**

- **Compound 2 (stigmastérol) and 3 (*β*-sitostérol)**

Mixture of **Stigmasterol,** C_29_H_48_O, (m/z 412) **and *β*-sitostérol,** C_29_H_50_O; (m/z 414). White powder. **Compound 2 (stigmastérol)**: RMN ^13^C (CDCl_3_, 150 MHz): 12.2 (C-29), 12.5 (C-24), 19.1 (C-28), 19.5 (C-27), 20.1 (C-26), 21.5 (C-11), 24.4 (C-15), 29.4 (C-16), 29.7 (C-25), 31.7 (C-7 et C-8), 31.9 (C-2), 36.5 (C-10), 37.2 (C-1), 39.7 (C-12), 40.6 (C-18), 42.2 (C-13), 42.3 (C-4), 50.2 (C-19), 51.2 (C-9), 55.9 (C-17), 56.8 (C-14), 71.8 (C-3), 121.7 (C-6), 129.3 (C-21),  129.2 (C-23), 138.4 (C-22), 138.5 (C-20) and 140.9 (C-5) (De-Ekmamkul et Potduang, 2003). **Compound 3 (*β*-sitostérol)**: RMN ^13^C (CDCl_3_, 150 MHz): 12.2 (C-29), 12.5 (C-24), 19.1 (C-28), 19.5 (C-27), 20.1 (C-26), 21.5 (C-11), 22.7 (C-23), 24.4 (C-15), 29.4 (C-16), 25.4 (C-22), 29.7 (C-25), 31.7 (C-7 et C-8), 31.9 (C-2), 36.5 (C-10), 37.2 (C-1), 39.7 (C-12), 40.6 (C-18), 42.2 (C-13), 42.3 (C-4), 50.2 (C-19), 51.2 (C-9), 55.9 (C-17), 56.8 (C-14), 71.8 (C-3), 121.7 (C-6), 129.3 (C-21),  138.5 (C-20) and 140.9 (C-5) [9]

^13^C RMN (CDCl_3_, 150 MHz) of compound **2** and **3**

^1^H RMN (CDCl_3_, 600 MHz) of compound **2** and **3**

- **Compound 4 (*****β*-sitostérol 3-*O*-*β*-D-glucopyranoside)**

***β*-sitostérol 3-*O*-*β*-D-glucopyranoside,** C_35_H_60_O_6_; (m/z 576). White powder.^13^C NMR (CDCl3): 11.7, 11.9, 18.8, 19.0, 19.2, 19.8, 21.1, 23.2, 24.3, 26.1, 28.3, 29.2, 30.0, 31.8, 31.9, 34.0, 36.2, 36.7, 37.2, 39.1, 39.7, 42.2, 45.8, 50.1, 56.0, 56.6, 62.6, 71.4, 75.1, 77.8, 78.4, 102.4, 121.7, 140.7. ^1^H NMR (C5D5N): 5.25 (1H, d, J = 4.6 Hz), 4.97 (1H, d, J = 7.7 Hz), 4.48 (1H, dd, J = 2.1, 11.7 Hz), 4.33 (1H, dd, J = 5.2, 11.7 Hz), 4.23 (1H, m), 4.21 (1H, m), 3.97 (1H, pseudo-t, J = 8.20 Hz), 3.90 (1H, m), 3.85 (1H, m), 0.55 (3H, s), 0.83 (3H, s), 0.88 (3H, d, J = 6.4 Hz), 0.76 (3H, d, J = 6.89 Hz), 0.77 (3H, d, J = 7.8 Hz), 0.80 (3H, t, J = 7.6 Hz).

**
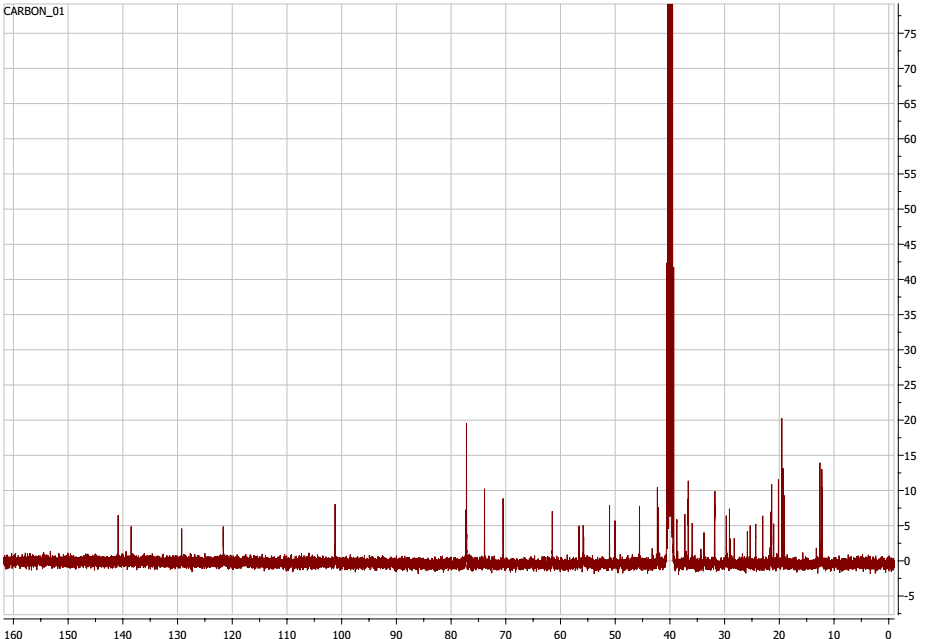
**

^13^C RMN (DMSO, 100 MHz) of compound **4**


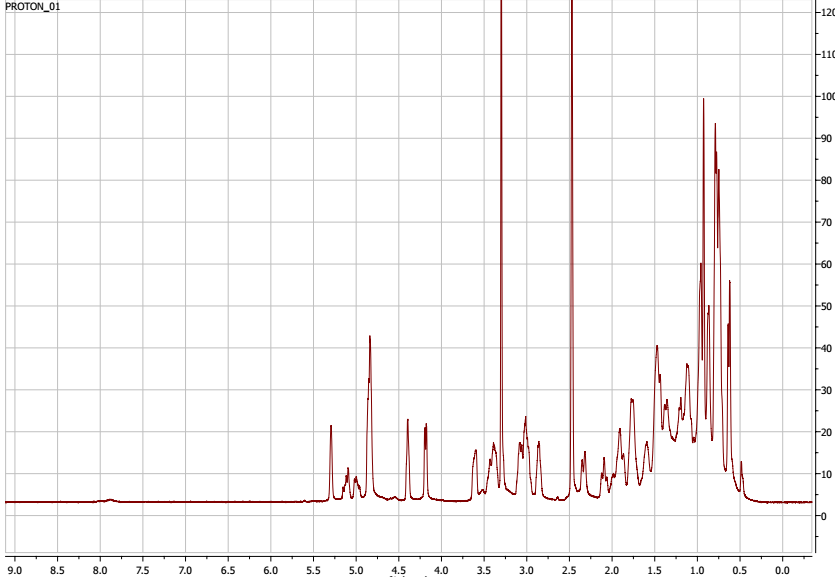
^1^H RMN (DMSO, 400 MHz) of compound **4**

- **Compound 5 (palmatin)**

**Palmatin**, C_21_H_22_NO_4_^+^; (m/z 352). Yellow powder. ^13^C NMR (CD_3_OD, 125 MHz): *δ* (ppm) 152.5 (C-9), 150.6 (C-3), 149.4 (C-2), 145.1(C-8), 144.3 (C-10), 138.4 (C-13a), 133.9 (C-4a), 128.7 (C-12a), 126.7 (C-11), 123.1 (C-12), 121.9 (C-1a), 119.9 (C-13), 119.1 (C-8a), 110.8 (C-4), 108.6 (C-1), 61.2 (9-OCH_3_), 56.3 (2-OCH_3_), 55.9 (C-6), 55.6 (3-OCH_3_), 55.3 (10-OCH_3_), 26.6 (C-5).^1^H NMR (CD_3_OD, 500 MHz): *δ* (ppm) 9.79 (1H, s, H-8), 8.83 (1H, s, H-13), 8.15 (1H, d, *J*=10.0 Hz, H-11), 8.05 (1H, d, *J*= 10.0 Hz, H-12), 7.70 (3H, s, H-1), 7.07 (1H, s, H-4); 4.93 (2H, d, *J*= 5.0 Hz, H-6); 4.22 (3H, s, H-9-OCH_3_); 4.13 (3H, s, H-2-OCH_3_); 4.01 (3H, s, H-3-OCH_3_); 3.95 (3H, s, H-10-OCH_3_); 3.29 (2H, d, *J*= 5.0 Hz, H-5).


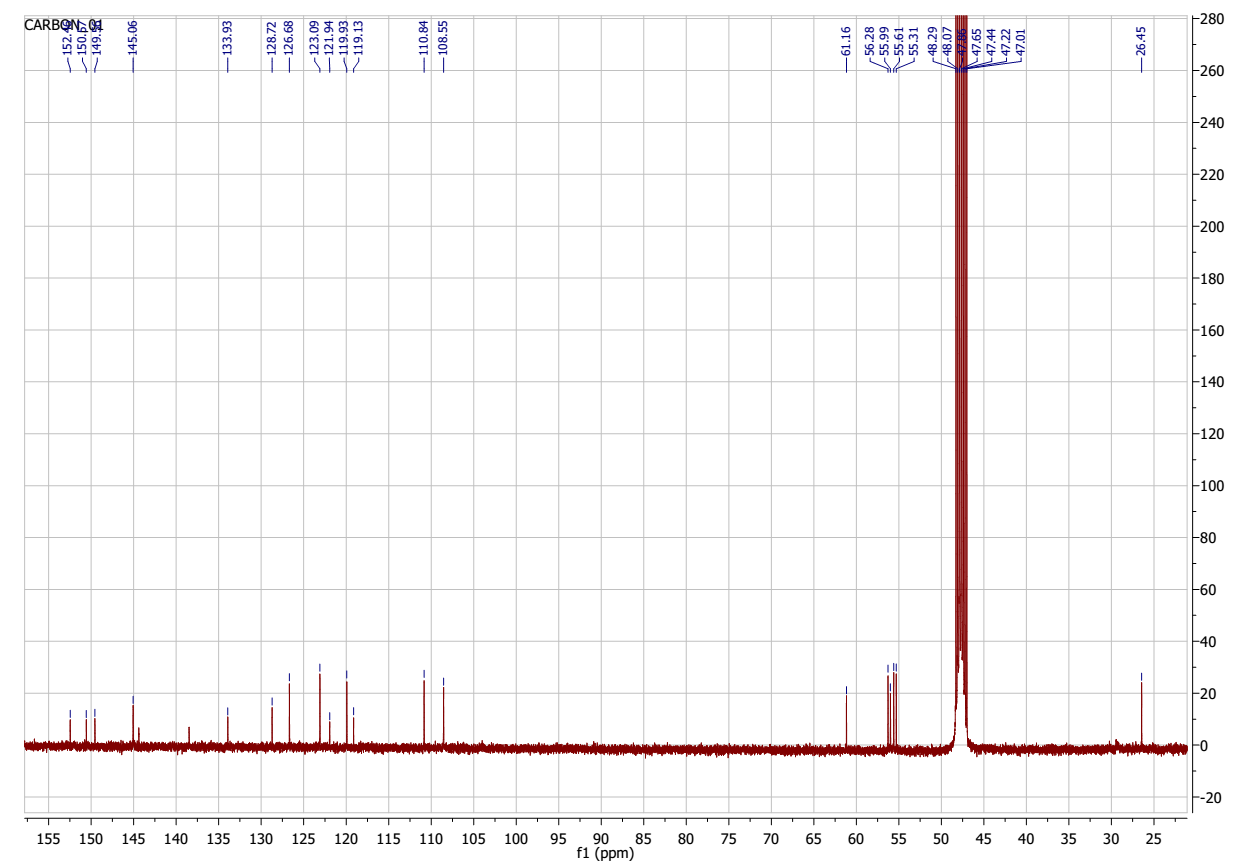


^13^C RMN (CDCD_3_, 125 MHz) of compound **5**


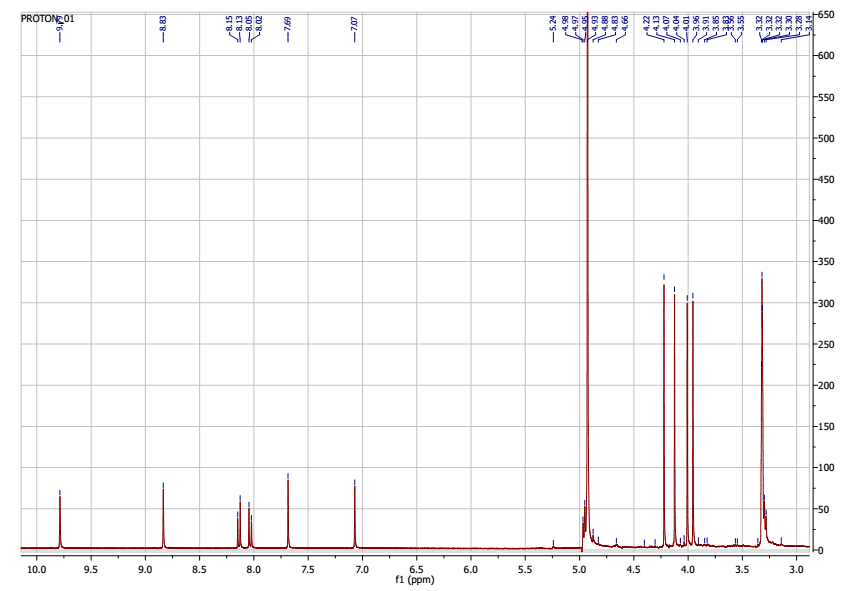


^1^H RMN (CDCD_3_, 500 MHz) of compound **5**

- **Compound 6 (homomangiferin)**

**Homomangiferin,** C_20_H_20_O_11_; (m/z 436). Yellow powder. ^13^C NMR (DMSO, 125 MHz): *δ* (ppm) 179.5 (C-9), 164.3 (C-1), 162.2 (C-3), 156.6 (C-4a), 154.6 (C-6), 151.2 (C-10a), 144.2 (C-7), 112.1 (C-8a), 108.4 (C-2), 108.1 (C-8), 103.0 (C-5), 101.7 (C-9a), 93.8 (C-4), 82.1 (C-5’), 79.4 (C-3’), 73.5 (C-1’), 71.1 (C-4’), 70.6 (C-2’), 61.9 (C-6’).^1^H NMR (DMSO, 500 MHz): *δ* (ppm) 7.35 (1H, s, H-8), 6.85 (1H, s, H-5), 6.35 (1H, s, H-4), 4.53 (1H, d, *J*= 10.0 Hz, H-1’), 4.14 (1H, t, H-2’), 3.70 (1H, d, *J=* 15 Hz, H-6’); 3.30 (1H, s, H-6’); 3.25 (1H, m, H-3’); 3.20 (1H, m, H-5’); 3.15 (1H, m, H-4’).


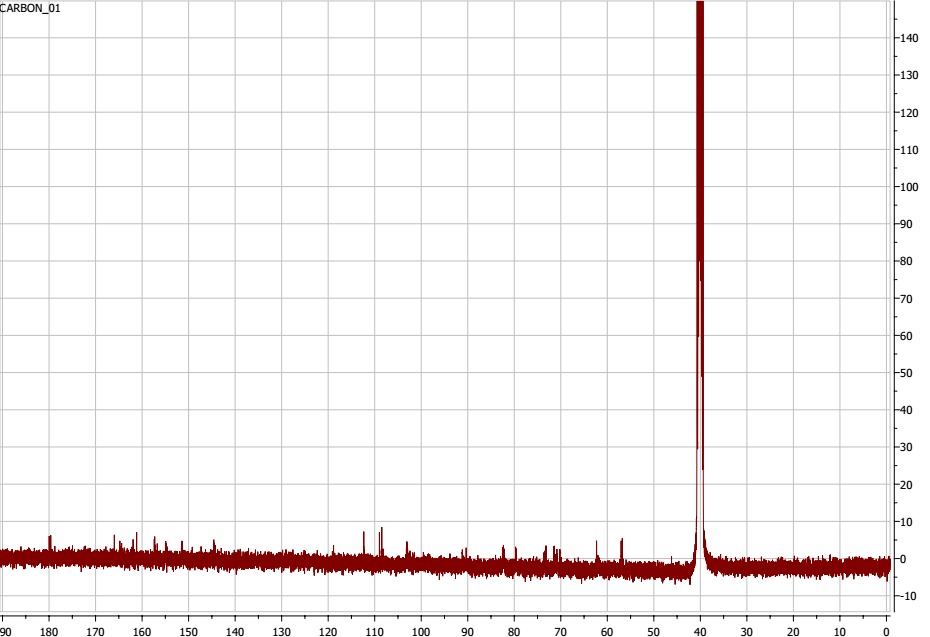


^13^C RMN (DMSO, 125 MHz) of compound **6**


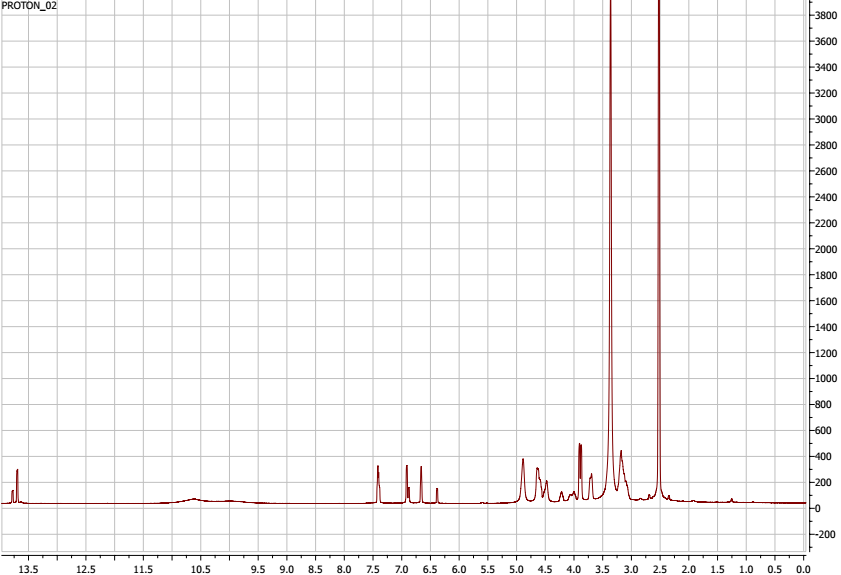


^1^H RMN (DMSO, 500 MHz) of compound **6**

- **Compound 7 (mangiferin)**

**Mangiferin,** C_19_H_18_O_11_; (m/z 422). Yellow powder. ^13^C NMR (DMSO, 100 MHz): *δ* (ppm) 179.7 (C-9), 164.4 (C-1), 162.2 (C-3), 157.3 (C-4a), 154.9 (C-6), 151.5 (C-10a), 144.4 (C-7), 112.4 (C-8a), 108.9 (C-2), 108.4 (C-8), 103.4 (C-5), 102.9 (C-9a), 91.1 (C-4), 82.2 (C-5’), 79.7 (C-3’), 73.4 (C-1’), 71.4 (C-4’), 70.8 (C-2’), 62.3 (C-6’), 57.1 (C-3-OCH_3_).^1^H NMR (DMSO, 400 MHz): *δ* (ppm) 7.42 (1H, s, H-8), 6.90 (1H, s, H-5), 6.66 (1H, s, H-4), 4.62 (1H, sl, H-1’), 4.21 (1H, s, H-2’), 3.70 (1H, s, H-6’), 3.36 (1H, s, H-6’), 3.20 (1H, m, H-3’), 3.17 (1H, m, H-5’), 3.18 (1H, m, H-4’).


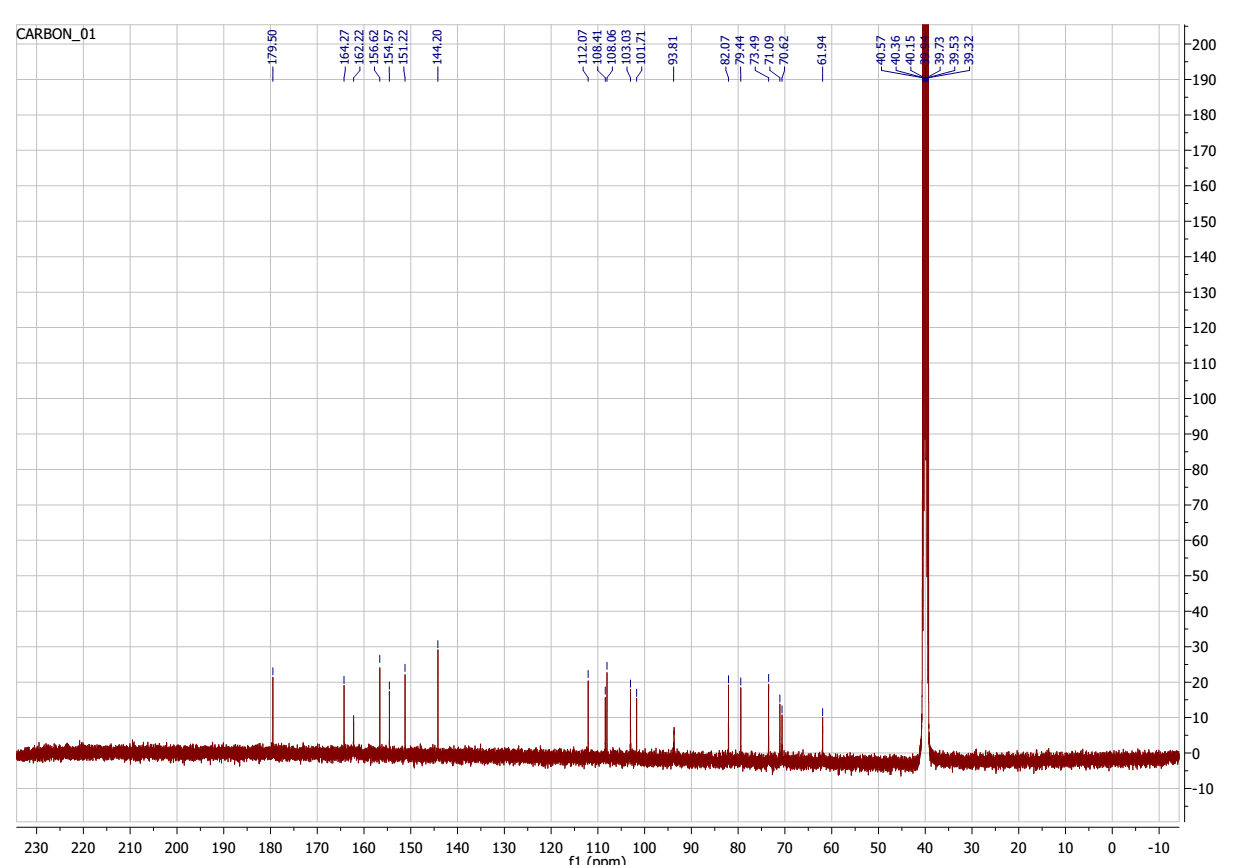


^13^C RMN (DMSO, 400 MHz) of compound **7**


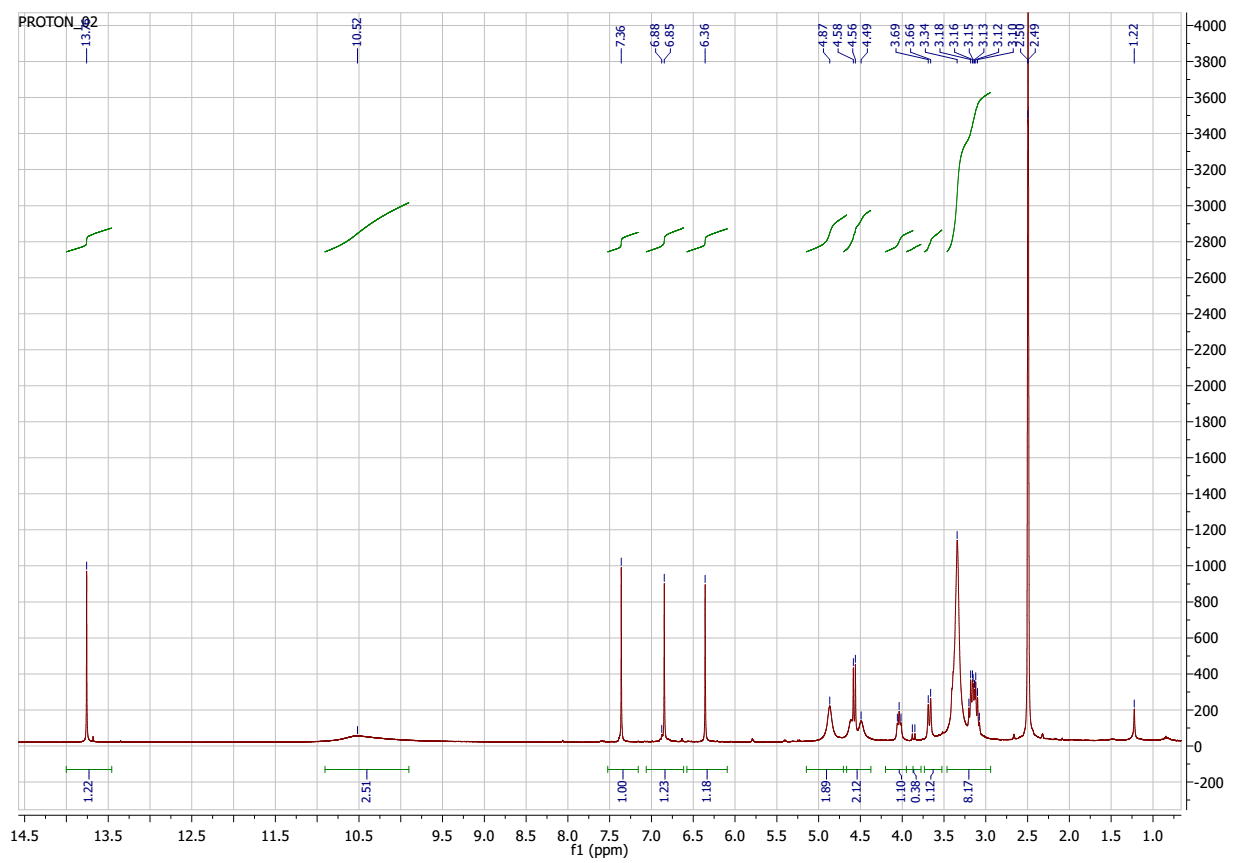


^1^H RMN (DMSO, 100 MHz) of compound **7**

***S3.*** *Preliminary evaluation of antibiotic-resistance modulatory activity of selected samples at sub-inhibitory concentrations against Pseudomonnas aeruginosa PA124*

| **Isolated compounds** | | **MIC (µg/mL) of antibiotics in the presence and absence of compounds with FIC in brackets** | | | | | | | | |
| --- | --- | --- | --- | --- | --- | --- | --- | --- | --- | --- |
|  |  | **CHL** | **CIP** | **ERY** | **GEN** | **KAN** | **NOR** | **PEN** | **STR** | **TET** |
|  | **()** | **>64** | **2** | **>32** | **>4** | **0,5** | **>16** | **>128** | **>32** | **8** |
| **1+2** | CMI/2 | >64 | 0,5 (0,25)S | 32 (0,5)S | 4 (0,5)S | <0,125(0,25)S | <0,125(0,007)S | >128 | 32 (0,5)S | 4 (0,5)S |
|  | CMI/4 | >64 | 0,5 (0,25)S | 32 (0,5)S | 4 (0,5)S | <0,125(0,25)S | <0,125(0,007)S | >128 | 32 (0,5)S | 4 (0,5)S |
|  | CMI/8 | >64 | 2 | >32 | >4 | 0,5 (1)I | >16 | >128 | >32 | 8 (1)I |
|  | CMI/16 | >64 | 2 | >32 | >4 | 0,5 (1)I | >16 | >128 | >32 | 8 (1)I |
| **4** | CMI/2 | >64 | 2 (1)I | 32 (0,5)S | 4 (0,5)S | <0,125(0,25)S | 8(<0,5)S | >128 | 32 (0,5)S | 4 (0,5)S |
|  | CMI/4 | >64 | 2 (1)I | 32 (0,5)S | 4 (0,5)S | <0,125(0,25)S | 8(<0,5)S | >128 | 32 (0,5)S | 4 (0,5)S |
|  | CMI/8 | >64 | 2 (1)I | >32 | >4 | 0,5 (1)I | >16 | >128 | >32 | 8 (1)I |
|  | CMI/16 | >64 | 2 (1)I | >32 | >4 | 0,5 (1)I | >16 | >128 | >32 | 8 (1)I |

^a^Antibiotics [ CIP: Ciprofloxacin, ERY: Erythromycin, GEN: Gentamycin, KAN: Kanamycin, NOR: Norfloxacin, STR: Streptomycin, TET: Tetracyclin]. S: Synergy; I: Indifference; ( ) : FIC (Fractional Inhibitory Concentration) of the antibiotics after association with compounds , 0 : MIC of the antibiotic alones.

**References**

1. Viveiros M, Jesus A, Brito M, et al. Inducement and reversal of tetracycline resistance in E*scherichia coli* K-12 and expression of proton gradient-dependent multidrug efflux pump genes. *Antimicrob Agents Chemother. 2005;* 49( 8): 3578-3582.

2. Elkins AC, Mullis BL. Substrate competition studies using whole-cell accumulation assays with the major tripartite multidrug efflux pumps of *Escherichia coli*. *Antimicrob Agents Chemother. 2007;* 51(3):923-929.

3. Mallea M, Mahamoud A, Chevalier J, et al. Alkylaminoquinolines inhibit the bacterial antibiotic efflux pump in multidrug-resistant clinical isolates. *Biochem J. 1998;* 376(3):801-805.

4. Mallea M, Chevalier J, Bornet C, et al. Porin alteration and active efflux: two in vivo drug resistance strategies used by *Enterobacter aerogenes*. *Microbiology. 2000;* 144 (11): 3003-3009.

5. Ghisalberti D, Masi M, Pages MJ, Chevalier J. Chloramphenicol and expression of multidrug efflux pump in *Enterobacter aerogenes.* *Biochem Biophys Res Commun. 2005;* 328 ( 4): 1113-1118.

6. Chevalier J. Pages MJ, Eyraud A, Mallea M. Membrane permeability modifications are involved in antibiotic resistance in *Klebsiella pneumoniae*. *Biochem Biophys Res Commun.2000;* 274(2):496-499.

7. Tran TQ, Mahendran RK, Hajjar E, et al. Implication of porins in beta-lactam resistance of *Providencia stuartii.*  *J Biol Chem.2010;* 285(42): 32273-32281.

8. Lorenzi V, Muselli A, Bernardini FA, et al. Geraniol restores antibiotic activities against multidrug-resistant isolates from gram-negative species. *Antimicrob Agents Chemother. 2009;*53(5):2209-2211.
